# The Effectiveness of Protected Areas in Conserving Globally Threatened Western Tragopan *Tragopan melanocephalus*

**DOI:** 10.3390/ani11030680

**Published:** 2021-03-04

**Authors:** Muhammad Naeem Awan, Jonas Geldmann, Francis Buner, Zafeer Saqib, Arshid Pervez, Qaisar Mahmood, Abeer Hashem, Al-Bandari Fahad Al-Arjani, Abdulaziz A. Alqarawi, Elsayed Fathi Abd_Allah, Tahir Ali Akbar

**Affiliations:** 1 Department of Environmental Sciences, COMSATS University, Abbottabad Campus, KPK, Islamabad 22010, Pakistan; pervez@cuiatd.edu.pk; 2 Conservation Science Group, Department of Zoology, University of Cambridge, Downing St., Cambridge CB2 3EJ, UK; jgeldmann@sund.ku.dk; 3 Center for Macroecology, Evolution and Climate, Globe Institute, University of Copenhagen, DK-2100 Copenhagen, Denmark; 4 Game and Wildlife Conservation Trust, Burgate Manor, Fordingbridge SP6 1EF, UK; fbuner@gwct.org.uk; 5 GIS & Ecoinformatics Laboratory, Department of Environmental Science, International Islamic University, Islamabad 46000, Pakistan; zafeer@iiu.edu.pk; 6 Botany and Microbiology Department, College of Science, King Saud University, Riyadh 11451, Saudi Arabia; habeer@ksu.edu.sa (A.H.); aarjani@ksu.edu.sa (A.-B.F.A.-A.); 7 Mycology and Plant Disease Survey Department, Plant Pathology Research Institute, ARC, Giza 12511, Egypt; 8 Plant Production Department, College of Food and Agricultural Sciences, King Saud University, Riyadh 11451, Saudi Arabia; alqarawi@ksu.edu.sa (A.A.A.); eabdallah@ksu.edu.sa (E.F.A.); 9 Department of Civil Engineering, COMSATS University, Abbottabad Campus, KPK, Islamabad 22010, Pakistan; drtahir@cuiatd.edu.pk

**Keywords:** western tragopan, protected areas management, conservation, western himalaya, Pakistan

## Abstract

**Simple Summary:**

Western Tragopan is a globally threatened pheasant species of the Western Himalayan Biodiversity Hotspot, whereas protected areas are tools used to protect species and their habitat. In this study, we selected protected areas falling within the potential habitat of the Western Tragopan and evaluated their management effectiveness to understand their role in the protection of the pheasants of global conservation concern. Our results show that only Machiara National Park scored just above 40% (indicating relatively weak management), 22 of the PAs fell within the 25–50% quantile (indicating weak management), and 3 scored below 25% (indicating poor management). PAs within the species distributional range covered 92,387 ha which is only 2% of the total potential habitat of the tragopan. Thus, we concluded that protected areas are not sufficiently contributing to protecting species and its habitat and need to revise their plans. We further recommended establishment of more protected areas within the potential habitat of the species to help protect this iconic species of Western Himalaya.

**Abstract:**

Protected areas are a critical tool to conserve biodiversity in the face of the global crisis of species extinction. Here, we present the first ever management effectiveness assessment of Pakistan’s Protected Areas (PAs). We link these assessments to the delivery of conservation outcomes focusing on the threatened Western Tragopan (*Tragopan melanocephalus*) endemic to Pakistan and India. We used two approaches, first mapping the spatial distribution of potential habitat coverage using machine learning ensemble models and second, an assessment of the management effectiveness of protected areas. Our results show that only Machiara National Park scored just above 40% (indicating relatively weak management), 22 of the PAs fell within the 25–50% quantile (indicating weak management), and 3 scored below 25% (indicating poor management). PAs within the species distributional range covered 92,387 ha which is only 2% of the total potential habitat of the Tragopan. Scoring of Planning element was insufficient both in term of the site and species. Likewise, inputs (e.g., research and monitoring program, staff numbers, staff training, current budget, security of budget, and management after process) were also inadequate. Finally, we recommend the establishment of more protected areas within the species potential habitat and inclusion of species-specific plans in Pakistan’s PAs management.

## 1. Introduction

Nature across most of the globe has now been significantly altered by multiple human drivers, with the great majority of indicators of ecosystems and biodiversity showing rapid decline. Seventy-five percent of our planet’s land surface is now significantly altered [1]. Protected areas (hereafter PAs) are a critical tool to conserve biodiversity in the face of the global biodiversity crises resulting from the increasing impact of humans [2,3]. This has resulted in land-cover changes, unsustainable utilization of species, the spread of invasive species, climate change, and pollution, all causing biodiversity declines and the loss of key ecosystem services [1,4].

South Asia is one of the regions at the forefront of global population and economic growth. According to the United Nations, the human population has more than tripled between 1950 and 2009 in South Asia, from 473 million to 1.6 billion, and is projected to grow a further 41% by 2050 [4]. Similarly, Pakistan, the sixth most populated country on Earth, has one of highest population growth rates in the world [3]. The population of Pakistan grew from 31 million people in 1951 to about 185 million people in 2014 and the accompanying increased demand for natural resources is accelerating the loss of biodiversity and environmental degradation [5].

Perhaps the most important and far-reaching response to the biodiversity crisis has been the development of Protected Areas (PAs), of which more than 238,563 have now been designated with most areas on land, and collectively protect just over 20 million km^2^, equivalent to 14.9% of the earth’s land surface [6]. PAs have long been regarded as an important tool for maintaining habitat integrity and species diversity [7,8]. PAs are increasingly becoming final refuges for threatened species and natural ecosystem processes as deforestation imperils global biodiversity probably more than any other existing threat. PAs are generally considered effective at abating habitat conversion and biodiversity loss [9,10]. The success of PAs has generally been evaluated using measures such as the representativeness of PA networks in terms of their species diversity, or coverage of endemic and threatened species [9].

### History of Protected Areas in Pakistan

Prior to 1966, Pakistan took no significant steps towards establishing a PAs network but the continuing noticeable decline of wildlife during the 1950s and 1960s prompted the Government of Pakistan in 1967 to commission the World Wildlife Fund (WWF) to undertake extensive surveys of the status of the wildlife in the country and requirement of its conservation. This led the WWF to carry out a survey of the country’s wildlife resources and recommended measures to arrest their deterioration [11]. These included the establishment of a PAs system in the country which initially included six sites within IUCN management category II (i.e., National Park), 45 in category IV (i.e., managed nature reserve/wildlife sanctuaries), and 4 in category V (i.e., protected landscapes/seascapes) covering ≥1000 ha. This initiative was followed by the formation of the wildlife enquiry committee in 1968, which made further recommendations for the establishment of five National parks, 18 wildlife sanctuaries and 52 game reserves [11]. These recommendations have been substantially exceeded with 4 national parks, 44 wildlife sanctuaries, and 65 game reserves established by the year 1978 (IUCN, 1990). Currently, Pakistan has 157 PAs of which five are classified as national parks of IUCN category II, 62 as wildlife sanctuaries (category IV), 5 as protected landscapes/seascapes (category V), 2 as managed resource protected areas (Category VI), and 83 as unclassified areas [5].

The importance of PAs in safeguarding biodiversity is now enshrined in the Aichi Target 11 that forms part of the Strategic Plan for Biodiversity 2011–2020 of the Convention on Biological Diversity which Pakistan is a party to [3]. Science has already demonstrated the contribution of PAs to species coverage [9] and has developed methods for evaluating the management effectiveness of PAs [12]. So, in this study we look at role of PAs in the conservation of Western Tragopan, a red-listed Galliformes which is endemic to the Western Himalayan biodiversity hotspot. With a relatively small geographical range found only in northern Pakistan and north-western India, it is an extremely elusive pheasant occurring between 2400–3500 m.a.s.l. [13]. In Pakistan, it occurs in comparatively smaller pockets in the northern parts of the country, i.e., Pallas valley, Kaghan valley, and Azad Jammu and Kashmir [14]. The majority of Protected Areas in Pakistan were created unsystematically, even no criteria was set for their selection, and demarcated without considering any ecological basis [15].

Here, we present an assessment of the effectiveness of Pakistan’s PA network in safeguarding the Western Tragopan (*Tragopan melanocephalus*). Our analysis includes two sequential and interconnected steps. First, we develop a habitat suitability model to identify the PAs that are critical for the conservation of the Western Tragopan. Second, for all PAs with a suitable habitat (*n* = 26), we assessed their management effectiveness following the process of adapting the Management Effectiveness Tracking Tool (METT) for PAs [16]. Finally, we make recommendations about future management priorities for Pakistan’s PAs and for the conservation of globally threatened species such as the Western Tragopan.

## 2. Materials and Methods 

### 2.1. Habitat Suitability Data

**Figure 1:**
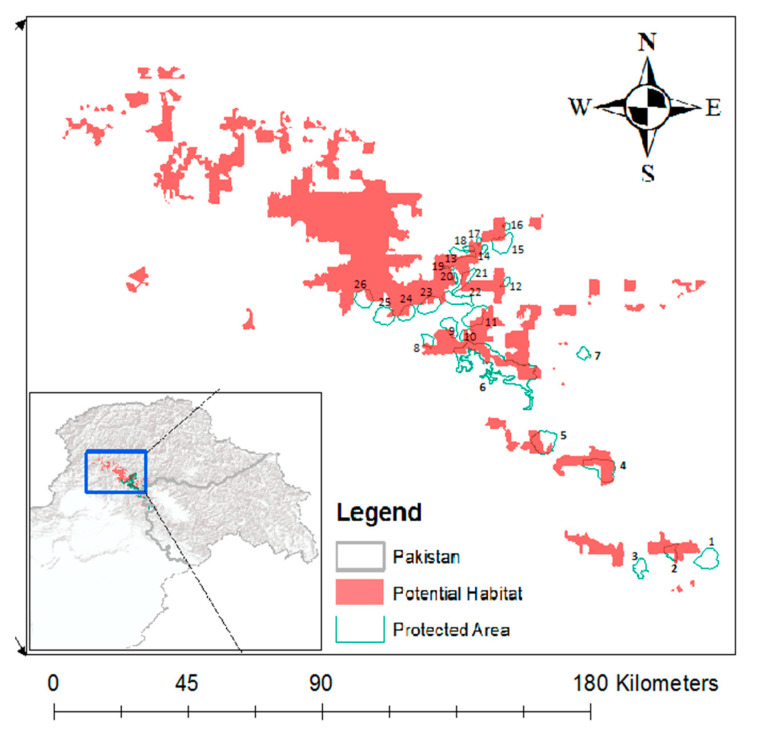
Spatial distribution of protected areas falling within the potential habitat of the species. For names of numbered protected areas see Table 1.

In order to predict the potential habitat of Western Tragopan in Pakistan, we first used data on breeding call count locations (*n* = 226) as a response variable against a suite of bioclimatic predictor variables. The breeding call count locations (*n* = 67) were GPS-marked during field surveys carried out for the period of 2008-2020. In order to make the study more comprehensive, records from previous studies [17] emphasizing three main distribution pockets in Pakistan were also added (*n* = 159, Figure 1). Second, we developed the habitat suitability maps by mapping the spatial distribution of the potential habitat coverage, modeled in software R for Windows Ver. 3.5.2; R (Core Team 2018) using the package ‘Dismo’ [18]. We choose three kinds of variables for our model that included bioclimatic variables (19 variables; https://www.worldclim.org/data/bioclim.html) and topographic and remote sensing. As the species is highly selective in altitude and aspects during the breeding season [14], so, some of variables related to topography were also considered in the model including elevation (elev), slope and transformed aspect value (aspv) based upon SRTM data in addition to Continuous Heat-Insolation Load Index (chin), Global ALOS landforms (alf), ALOS global topographic diversity (tdiv). [19]. We further used remote sensing derived normalized difference vegetation and snow indices (NDVI and NDSI) based upon cloud free median values of sentinel 2 satellite and resampled at 1 km resolution [20].

The machine learning models used for building an ensemble (average) of three analysis included Random Forest [21], Support Vector Machine [22], and Maximum Entropy Modeling (Maxent; [23]. We then built an average ensemble of the three models as the final potential habitat map of Western Tragopan in Pakistan.

### 2.2. Management Effectiveness Assessment 

Effectiveness assessment were undertaken using Management Effectiveness Tracking Tool (METT) [16] which builds on the WCPA Management effectiveness Framework (see [16] and is based on the idea that good protected area management follows a process that has six distinct stages, or elements: (1) it begins with understanding the context of existing values and threats, (2) progresses through planning, (3) allocation of resources (inputs), (4) result of management actions (processes), and eventually produces (5) products and services (outputs) and (6) impacts or outcomes (Supplementary File S1) [16]. Furthermore, we used a threat assessment sheet to evaluate and quantify different threats to Pakistan’s PAs generally and in regard to Western Tragopan specifically (Supplementary File S2) [24].

The METT data was collected through five consultative workshops, with 15 participants each taking place between March and June 2020. Participants were selected from people primarily working in PAs or directly/indirectly involved with PAs including PA managers and staff (*n* = 10), students or researchers (*n* = 2) and local representatives (*n* = 3). Each participant only participated in one workshop. In this way, 26 PAs with known Western Tragopan occurrence were evaluated, including one national park (Machiara), six game reserves, one wildlife sanctuary and 18 with other designation types (Figure 1, Table 1).

A questionnaire was used to collect data on some basic information about the site, such as name, size and location. We used a unique site code given to the protected area included from the World Database on Protected Area (WDPA) accessed via the UNEP-World Conservation Monitoring Centre website at: www.unep-wcmc.org/wdpa. Other contextual information such as local designation, i.e., National park, nature reserve etc., along with the IUCN protected area management category [13] ownership, staff number, and budget were also recorded.

For the 30 specific questions in the METT, the assessment was made by assigning a score ranging between 0 (poor or absent) to 3 (excellent or fully implemented). Four answers were provided against each question to help assessors to make judgments as to the level of score given. In addition, supplementary questions were used to elaborate on key themes in the previous questions and provide additional information and points (see SF). For threat analysis a separate sheet was used to evaluate the different types of threats to the species and its habitat within each protected area. Each sheet was holding questions about a set of 12 categories of threats as described in the Management Effectiveness Tracking Tool (METT) by [16] following the taxonomy laid out in [25]. Each category holds relevant threats which were scored according to the intensity from low to high. 

### 2.3. Data Analysis 

Using a Geographical Information System (GIS)-based habitat suitability analysis of key habitat variables, we calculated the potential habitat suitable for Western Tragopan in Pakistan (Figure 1). We then mapped the boundary of the PAs to estimate the potential habitat of the species falling within the PAs and outside the PAs. 

The overall management effectiveness scores were used to understand the management effectiveness at each protected area and across the network [19]. Similarly, scores were also used to evaluate the threat level in all PAs whereas species specific threats were also scored to underhand scenario of threats to PAs and species. We calculated the mean value of each variable with Standard Error (SE), percentage value of each question and further calculated mean ± SE for each element of the WCPA framework. Finally, to understand the correlation among different variables, we examined the coefficients of determination (Pearson correlation) between different variables of the contributing elements of Protected Areas Management effectiveness and threats.

## 3. Results

### 3.1. Spatial Distribution of Protected Areas

We present here the METT assessments from all 26 PAs located within the potential habitat of the Western Tragopan in Pakistan (Table 1). 

Within the Pakistani Himalayas, the PAs network falling within the Western Tragopan’s distributional range cover 92,387 ha corresponding to only 2% of the total potential habitat of the tragopan (Figure 1). Seventeen protected areas (65%) fall within the boundary of province of Khyber Pakhtunkhawa and only seven (35%) in the state of Azad Jammu and Kashmir (Figure 1). Inside the PAs about 50% (47,468 ha) of the landscape is the potential habitat of the Western Tragopan, whereas approximately the same landscape portion (50%) within PAs is not suitable for the species.

### 3.2. Protected Areas Management Effectiveness Analysis 

#### 3.2.1. Overall Ranking of the Contributing Protected Areas 

**Figure 2:**
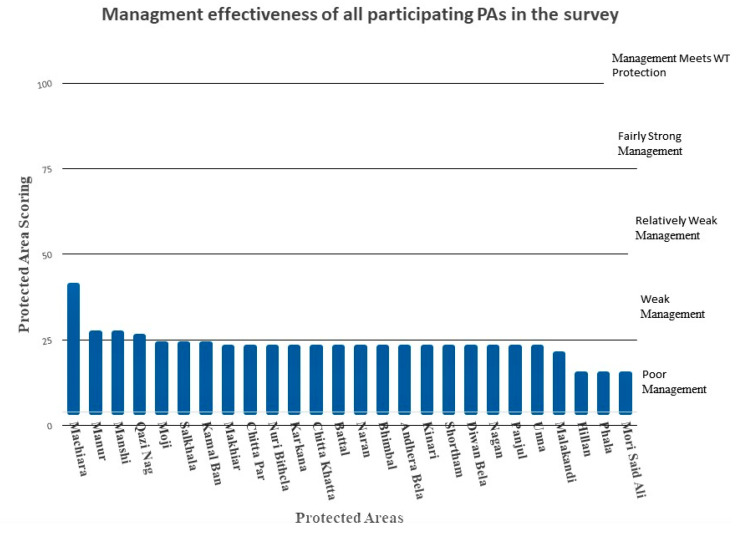
Management effectiveness of different protected areas surveyed during the study.

All 26 PAs reported severe deficits in their management. Only one (Machiara National Park) scored close to 50%, when all questions were combined, while 22 PAs fell within the 25–50% quantile, indicating that they are weakly managed, and three scored less than 25% when looking at the scores across all questions (Figure 2). 

All 26 protected areas showed severe deficiencies in resources and management capacities (As represented by the combined METT score across all questions; Figure 2). Of the 26 PAs, Machiara National park was the highest ranked (although still within the weak management category), scoring 41% (mean = 1.7, S.E. = 2.1) followed by Manur (27%), Manshi (27%), and Qazi Nag (26%) which scored mean 1.1, S.E. = 0.17, mean 1.1, S.E. = 0.18, and mean 1.1, S.E. = 2.1, respectively. The least scoring PAs are Hillan, Phala, and Mori Said Ali with 15% each, (mean = 0.6, S.E. = 0.16). 

#### 3.2.2. Scoring Based on the Elements of the WCPA Framework

**Figure 3:**
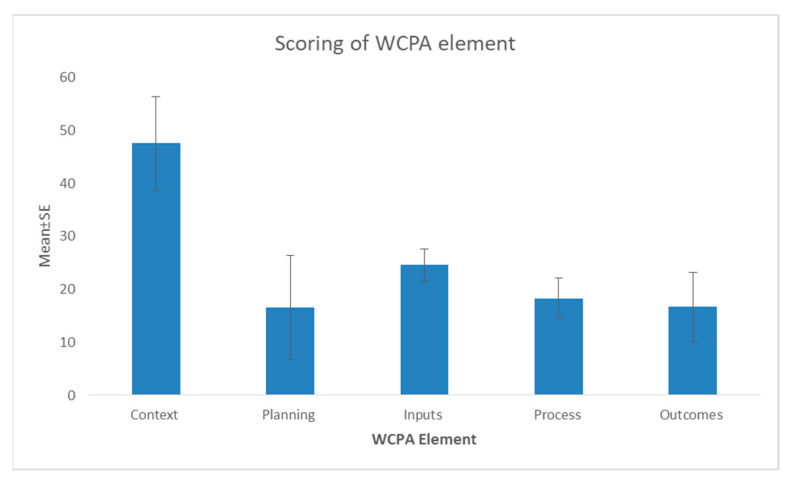
Scoring results of different elements of WCPA from participating protected areas in Pakistan.

Dividing the scores by the six elements of the WCPA management effectiveness framework ([16] revealed some interesting differences. PAs, on average, were recorded as reasonably effective for questions related to their context (mean = 47.6, S.E. = 8.846). Thus, the PAs were legally recognized, had clear boundary demarcation, as well as clear biodiversity resource inventories and management objectives (Figure 3). However, for other elements, the results were less encouraging. Planning was insufficient both in term of the site and species (mean = 16.6, S.E. = 9.795). Likewise, inputs (e.g., research and monitoring program, staff numbers, staff training, current budget, security of budget, (mean = 24.6, S.E. = 3.01), and management process (mean 18.33, S.E. = 3.76) were also inadequate (Figure 3).

#### 3.2.3. Target Species Management in and Outside Protected Areas

While having adequate resources and well-established management systems is key, these are ultimately a means to an end—delivering positive conservation outcomes. To address this, we also had four questions that addressed the Pas’ contribution to maintaining and/or improving the conservation status of the Western Tragopan—our target species. Overall, these questions revealed that the Western Tragopan is poorly managed (mean = 1.30, S.E. = 0.15). In the planning element there were two species specific questions: (1) Species specific action plan and (2) planning outside of the PA for the target species. Both questions scored zero indicating that all protected areas are lacking species specific action plans and no planning outside the PA to help to protect the species (Supplementary Files S1 and S2).

#### 3.2.4. Ecological Outcomes

**Figure 4:**
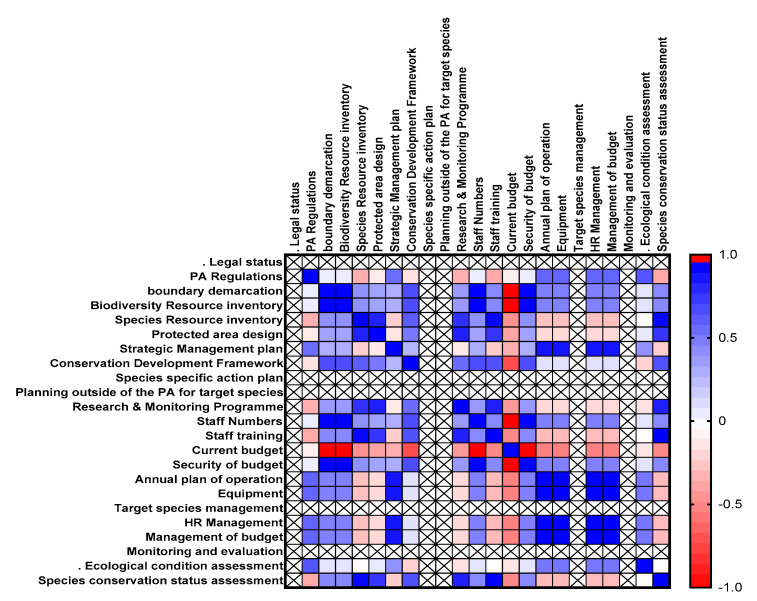
Correlation matrix between different variables of the contributing elements of PAME. Each line and column represent one of the questions in the Management Effectiveness Tracking Tool (METT). The color indicates the correlation with zero showing no correlation between the two questions, one (blue) showing a perfect positive correlation and minus one (red) a perfect negative correlation.

The results of the ecological outcomes were equally ineffective compared to the species outcomes (mean = 16.66, S.E. = 6.56). The survey included three questions about the state of the ecological outcome all showing that the PAs on average had a poor ecological status: (1) ecological condition assessment (mean = 0.77, S.E. = 0.08, 25.6%), (2) species conservation status assessment (mean = 0.15, S.E. = 0.07, 5.1%), and (3) species protection systems (mean = 1.00, S.E. = 0.00, 33.3%) (Figure 4).

The current PAs’ budgets (inputs) showed a positive correlation with the species’ protection system and ecological condition assessment (outcomes, *p* < 0.0001) but a negative, though not significant, correlation with the species’ conservation status assessment (*p* = 0.1250) suggesting that PAs with more adequate budgets also had higher scores for conservation outcomes. Similarly, a Strategic Management Plan included in the planning element resulted in a positive correlation with the ecological condition assessment (*p* < 0.05) and species protection system (*p* < 0.0001) but negative, though not significant, correlation with species conservation status assessment (*p* = 0.1250).

Ecological outcomes of the survey were found negatively correlated with most of the variables of contributing elements, e.g., ecological condition assessment is negatively correlated with PAs design (*p* = 0.66), whereas species conservation status assessment has also been recorded negatively correlated with Protected area design (*p* = 0.778), Species Resource inventory (*p* = 0.93), Conservation Development Framework (*p* = 0.68), Research and Monitoring program (*p* = 0.86) and Staff training (*p* = 1.00). 

### 3.3. Threats 

#### 3.3.1. Site-Wise Threat Ranking

**Figure 5:**
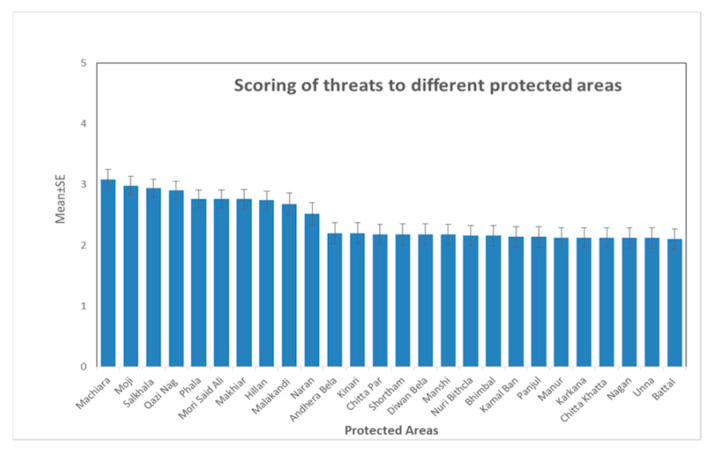
Threats scoring for participating protected areas.

Based on the results of our surveys, Machiara National park had the highest level of threats across all categories (mean= 3.08, S.E. = 0.16,). Three sites i.e., Moji, Salkhala, and Qazi Nag received mean = 2.98, S.E. = 0.15, mean = 2.94, S.E. = 0.15 and mean = 3.08, S.E. = 0.16, respectively. Batal received the lowest score, mean = 2.9, S.E. = 0.15 (Figure 5). 

**Figure 6:**
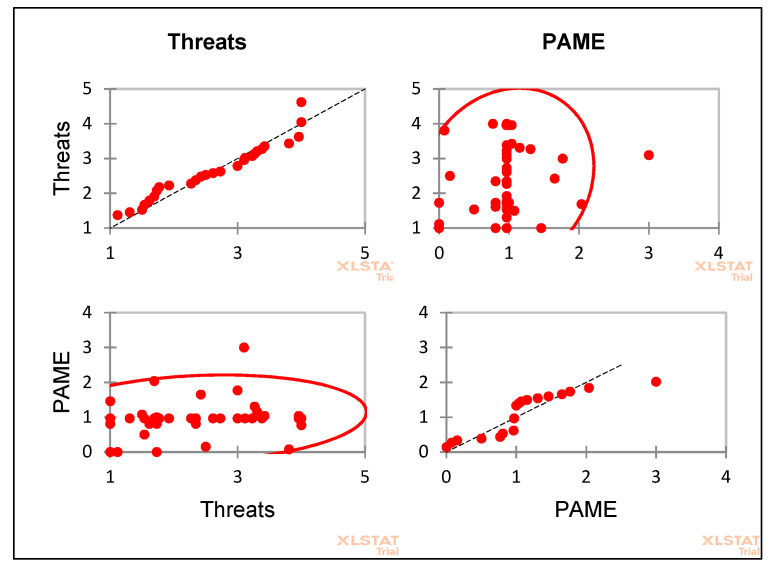
Correlation between Protected Areas Management and Threats.

There was a positive correlation between PAs management effectiveness and threats (*p* < 0.0001) (Figure 6). 

#### 3.3.2. Specific Threat’s Ranking (Species Related)

**Figure 7:**
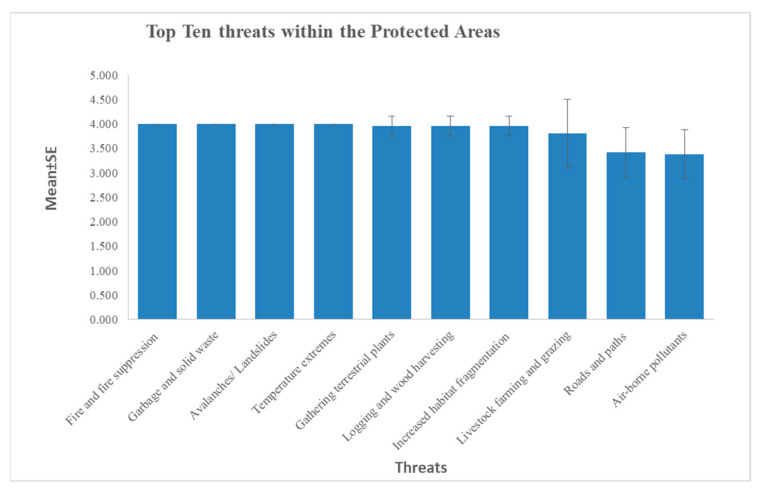
Dynamics of major threats among 26 PAs in Pakistan where Western Tragopan occurs.

The results of surveys show that the conservation concerns related to fire and fire suppression, garbage and solid waste, avalanches/landslides, and temperature extremes are the main threats facing all protected areas, with all 26 PAs achieving the top score (mean = 4.00, S.E. = 0.00). Furthermore, species specific threats recorded were habitat fragmentation (mean =3.96, S.E. = 0.20), livestock farming and grazing (mean = 3.81, S.E. = 0.141), roads and paths (mean = 3.42, S.E. = 0.10) hunting, killing and collecting of terrestrial animals (mean = 3.31, S.E. = 0.09), housing and settlement (mean = 3.11, S.E. = 0.09), natural deterioration (mean = 3.00, S.E. = 0.00), gathering of terrestrial plants (mean = 3.96, S.E. = 0.20), and logging and wood harvesting (mean = 3.96, S.E. = 0.20, Figure 7).

## 4. Discussion

To understand whether national PAs networks are meeting the government’s obligations towards the Convention on Biological Diversity it is critical to understand the strengths and needs of a protected area system, identify the best practice, and keep countries accountable for their obligations of maintaining an effective network of PAs. While our results show that most PAs in Pakistan do not sufficiently cover the core habitats of the Western Tragopan, our results also highlight the current gaps in the PAs network. These can only be filled if adequate resolution records of species-associated ecosystem distributions are scientifically incorporated [25,26]. Therefore, our maps of potential suitable habitat are of great help for the conservation planning of the Western Tragopan within the Pakistani part of its distributional range under PAs management. Systematically planned PAs always aim to ensure representative ecosystem protection to help retain threatened species habitats [8,25,26,27].

The results further provide evidence that the management of Pakistan’s PAs for Western Tragopan conservation is likely to be insufficient as it covers only 2% of the total potential habitat of the species in Pakistan (Figure 1). Whereas, according to Birdlife International [18] the largest global population of the Western Tragopan occurs in Palas Valley in northern Pakistan, the area lacks any PAs to help protect the species and associated ecosystem (see Figure 1). The gap in PA coverage is of particular concern when a species is at risk of extinction [10]. Hence, to capture a larger proportion of the species’ total population, it would be advisable to redesign and expand Pakistan’s PAs. This, in turn, would result in helping to safeguard the Western Tragopan together with other globally important Himalayan Galliformes sharing the same ecosystem [25,27].

When looking at the management effectiveness and maintenance of conservation outcomes, our results show that the PAs were the strongest in terms of understanding the context of protection while other elements showed severe inadequacies. This was especially the case for outcomes. This suggests that the existing PAs might have been designed appropriately to help conserve some red-listed species like Western Tragopan but lack the resources to actually do so. However, our results are based on assessments by local stakeholders who rarely can base these on high quality surveys. This may be because researchers mostly focused on monitoring iconic species and the most critical conservation threats, while long-term ecological monitoring has consequently been given little attention [25]. This is despite expert recommendation for increased focus on regular monitoring of Western Tragopan populations including robust research techniques such as radio telemetry [17,25]. Our results indicate that the management of Pakistan’s PAs is currently not adequate with all PAs ranking below the relatively weak management category. Management planning for the site as well as the species (e.g., research and monitoring program, staff numbers, staff training, current budget, security of budget) and affiliated management processes were also inadequate, all of which need strict consideration to improve the role of the PAs to improve the conservation the Western Tragopan, Pakistan’s national bird. Encouragingly, the ecological outcomes were positively correlated with PAs design, species conservation status assessment, species resource inventory, conservation development framework, research and monitoring programs, and staff training. These results mirror results found in previous studies outside Pakistan [27]. Thus, our results provide proof that PAs must be managed effectively to successfully protect species of global conservation concern such as the Western Targopan. Unfortunately, our results show that while a positive correlation was found, the absolute levels of management effectiveness are weak and likely entirely insufficient to maintain the species in perpetuity. 

PA networks in many countries do not adequately represent the highest priority areas for biodiversity [7] nor threatened species [28]. Our results provide a fitting example with the Machiara National Park, which ranked highest in the threats scoring while scoring highest in terms of management effectiveness. This provides an interesting pattern, potentially showing that what little funding exists for protected areas, is targeted to areas under highest pressure. Alternatively, this may also indicate the existing gaps in successfully addressing the threats for one of the most prestigious of Pakistan’s PAs where current levels of management appear not to effectively address existing threat levels. For effective conservation, PAs need adequate resources and effective management [29]. Systematically planned protected areas (PAs) aim to warrant characteristic samples of ecosystems are protected and threatened species’ habitats are reserved [8,28,30].

Birdlife International [18] already identified some threats for the Western Tragopan, such as habitat degradation and fragmentation, browsing of understory shrubs by livestock, tree-lopping for animal fodder and fuel wood-collection, disturbance by grazers. This study additionally highlights further threats inside PAs such as fire and fire suppression, garbage and solid waste management, avalanches/landslides and temperature extremes, in addition to the current main threats faced by all protected areas (Figure 5).

## 5. Conclusions

We conclude that current PAs management is not sufficiently effective in protecting the Western Tragopan and its habitat in Pakistan. We therefore recommend a major revision of all of Pakistan’s PAs management plans including specific targets for threatened species such as the Western Tragopan. Furthermore, management plans for the internationally recognized Important Bird and Biodiversity Areas [14] must be developed to help protect the species and its habitat also outside of the PAs, with priorities given to threatened species facing global extinction risk. The habitat model presented in the study provides a guideline for future research and monitoring and the establishment for further PAs which is expected to help to contribute to the protection of this species of global conservation concern together with the fragile ecosystem it inhabits. 

## Figures and Tables

**Table 1:** Protected areas falling within the potential habitat of the species in Pakistan.

Site Code	Name of the PAs (Area)	National Category	IUCN Category	Size (ha) Govt. Notified	Long	Lat
P1	Hillan (AJK)	Game Reserve	VI	384	4°15′18.47″ E	33°57′5.30″ N
P2	Phala (AJK)	Game Reserve	VI	472	74°10′7.80″ E	3°58′52.71″ N
P3	Mori Said Ali (AJK)	Game Reserve	VI	273	74° 4′28.82″ E	3°56′14.50″ N
P4	Qazi Nag (AJK)	Game Reserve	VI	4830	73°57′47.36″ E	34°13′22.82″ N
P5	Moji (AJK)	Game Reserve	VI	3859	73°47′15.69″ E	34°17′50.67″ N
P6	Machiara (AJK)	National Park	II	13,532	73°38′19.47″ E	34°31′53.90″ N
P7	Salkhala (AJK)	Game Reserve	IV	890	73°53′39.85″ E	34°33′2.87″ N
P8	Makhiar (KPK)	Reserve Forest	IV	1035	73°25′58.74″ E	34°35′15.51″ N
P9	Malakandi (KPK)	Reserve Forest	IV	1923	73°30′32.27″ E	34°36′36.21″ N
P10	Chitta Par (KPK)	Reserve Forest	IV	918	73°34′35.49″ E	34°36′59.33″ N
P11	Nuri Bithcla (KPK)	Reserve Forest	IV	1787	73°34′12.19″ E	4°38′29.64″ N
P12	Manur (KPK)	Reserve Forest	IV	425	73°38′59.00″ E	34°46′10.91″ N
P13	Karkana (KPK)	Reserve Forest	IV	1452	73°34′32.92″ E	34°50′11.92″ N
P14	Chitta Khatta (KPK)	Reserve Forest	IV	361	73°36′33.97″ E	34°51′38.08″ N
P15	Battal (KPK)	Reserve Forest	IV	2500	73°38′55.08″ E	34°52′41.20″ N
P16	Naran (KPK)	Reserve Forest	IV	290	73°39′41.74″ E	34°55′11.42″ N
P17	Bhimbal (KPK)	Reserve Forest	IV	220	73°35′23.85″ E	34°51′52.54″ N
P18	AndheraBela (KPK)	Reserve Forest	IV	410	73°33′29.65″ E	34°51′20.13″ N
P19	Kinari (KPK)	Reserve Forest	IV	241	73°29′18.43″ E	34°48′3.44″ N
P20	Shortham (KPK)	Reserve Forest	IV	272	73°30′36.66″ E	34°46′9.02″ N
P21	Diwan Bela (KPK)	Reserve Forest	IV	1510	73°31′5.70″ E	34°44′8.88″ N
P22	Kamal Ban (KPK)	Reserve Forest	IV	2212	73°31′35.14″ E	34°42′38.71″ N
P23	Manshi (KPK)	Wildlife Sanctuary	IV	2560	73°25′50.96″ E	34°42′17.99″ N
P24	Nagan (KPK)	Reserve Forest	IV	1637	73°22′40.98″ E	34°40′17.08″ N
P25	Panjul (KPK)	Reserve Forest	IV	2482	73°18′36.90″ E	34°40′11.70″ N
P26	Unna (KPK)	Reserve Forest	IV	2249	73°16′24.25″ E	34°43′23.30″ N
	Total Area			92,387		

## Supplementary Materials

The following are available online at https://www.mdpi.com/2076-2615/11/3/680/s1, File S1: Questionnaire for Assessment of Protected Areas Management Effectiveness in the conservation of Western Tragopan in Pakistan, File S2: Questionnaire for Assessment of Threats to Species and its habitat in PAs.

## References

[1] Díaz S., Settele J., Brondizio E.S., Ngo H.T., Guèze M., Agard J., Arneth A., Balvanera P., Brauman K.A., Butchart S.H.M., IPBES (2019). Summary for Policymakers of the Global Assessment Report on Biodiversity and Ecosystem Services of the Intergovernmental Science-Policy Platform on Biodiversity and Ecosystem Services.

[2] Bradshaw C.J., Brook B.W. (2014). Human population reduction is not a quick fix for environmental problems. Proc. Natl. Acad. Sci. USA.

[3] United Nations (2016). Sustainable Development Goals. https://sustainabledevelopment.un.org/topics/sustainabledevelopmentgoals.

[4] United Nations (2009). World Population Prospects: The 2008 Revision Population Database. http://esa.un.org/wup2009/unup/index.asp.

[5] Government of Pakistan (2015). Pakistan National Biodiversity Strategy and Action Plan. Islamabad. https://www.iucn.org/sites/dev/files/import/downloads/nbsap_1st_draft_23_3_15.pdf.

[6] UNEP-WCMC, IUCN, NGS (2018). Protected Planet Report 2018.

[7] Butchart S.H.M., Clarke M., Smith R.J., Sykes R.E., Scharlemann J.P.W., Harfoot M., Buchanan G.M., Angulo A., Balmford A., Bertzky B. (2015). Shortfalls and solutions for meeting national and global conservation area targets. Conserv. Lett..

[8] Coad L., Leverington F., Knights K., Geldmann J., Eassom A., Kapos V. (2015). Measuring impact of protected area management interventions: Current and future use of the Global Database of Protected Area Management Effectiveness. Philos. Trans. R. Soc. Lond. B. Biol. Sci..

[9] Rodrigues A.S.L., Akçakaya H.R., Andelman S.J., Bakarr M.I., Boitani L., Brooks T.M., Chanson J.S., Fishpool L.D.C., Da Fonseca G.A.B., Gaston K.J. (2004). Global gap analysis: Priority regions for expanding the global protected-area network. BioScience.

[10] Clark N.E., Boakes E.H., McGowan P.J.K., Mace G.M., Fuller R.A. (2013). Protected Areas in South Asia Have Not Prevented Habitat Loss: A Study Using Historical Models of Land-Use Change. PLoS ONE.

[11] Geldmann J., Barnes M., Coad L., Craigie I., Hockings M., Burgess N. (2013). Effectiveness of terrestrial protected areas in reducing biodiversity and habitat loss. Collab. Environ. Evid..

[12] Laurance W.F., Useche D.C., Rendeiro J., Kalka M., Bradshaw C.J.A., Sloan S.P., Laurance S.G., Campbell M., Abernethy K., Alvarez P. (2012). Averting biodiversity collapse in tropical forest protected areas. Nature.

[13] IUCN (1990). IUCN Directory of South Asian Protected Areas.

[14] BirdLife International (2020). Species Factsheet: Tragopan Melanocephalus. http://www.birdlife.org.

[15] Leverington F., Costa K.L., Pavese H., Lisle A., Hockings M. (2010). A Global Analysis of Protected Area Management Effectiveness. Environ. Manag..

[16] Stolton S., Hockings M., Dudley N., Mackinnon K., Whitten T., Leverington F. (2007). Management Effectiveness Tracking Tool. Reporting Progress at Protected Area Sites.

[17] Awan M., Buner F., Kingdon N. (2016). A review of published and unpublished surveys of a red-listed ‘flagship species’, the Western Tragopan Tragopan melanocephalus in Azad Jammu and Kashmir, Pakistan. Bird Conserv. Int..

[18] Fick S.E., Hijmans R.J. (2017). WorldClim 2: New 1-km spatial resolution climate surfaces for global land areas. Int. J. Climatol..

[19] Earth Engine Data Catalog. https://developers.google.com/earth-engine/datasets/tags/topography.

[20] The European Space Agency. https://sentinel.esa.int/.

[21] Breiman L. (2001). Random Forests. Mach. Learn..

[22] Kecman V., Wang L. (2005). Active-set methods for support vector machines. Support Vector Machines: Theory and Applications.

[23] Phillips S.J., Anderson R.P., Schapire R.E. (2006). Maximum entropy modelling of species geographic distributions. Ecol. Model..

[24] Salafsky N., Salzer D., Stattersfield A.J., Hilton-Taylor C., Neugarten R., Butchart S.H.M., Collen B.E.N., Cox N., Master L.L., O’Connor S. (2008). A Standard Lexicon for Biodiversity Conservation: Unified Classifications of Threats and Actions. Conserv. Biol..

[25] Barr L.M., Pressey R.L., Fuller R.A., Segan D.B., McDonald-Madden E., Possingham H.P. (2011). A new way to measure the world’s protected area coverage. PLoS ONE.

[26] Dunn J.C., Buchanan G.M., Stein R.W., Whittingham M.J., McGowan P.J. (2016). Optimising different types of biodiversity coverage of protected areas with a case study using Himalayan Galliformes. Biol. Conserv..

[27] Larsen F.W., Turner W.R., Mittermeier R.A. (2014). Will protection of 17% of land by 2020 be enough to safeguard biodiversity and critical ecosystem services?. Oryx.

[28] Symstad J., Chapin F.S., Wall D.W., Gross K.L., Huenneke L.F., Mittelbach G.G., Peters D.P.C., Tilman D. (2003). Longterm and large-scale perspectives on the relationship between biodiversity and ecosystem functioning. BioScience.

[29] Polak T., Watson J.E.M., Fuller R.A., Joseph L.N., Martin T.G., Possingham H.P., Venter V.O., Carwardine J. (2015). Efficient expansion of global protected areas requires simultaneous planning for species and ecosystems. R. Soc. Open. Sci..

[30] Nolte C., Agrawal A., Silvius K.M., Soares-Filho B.S. (2013). Governance regime and location influence avoided deforestation success of protected areas in the Brazilian Amazon. Proc. Natl. Acad. Sci. USA.

