# Supplementary material for: The Effectiveness of Protected Areas in Conserving Globally Threatened Western Tragopan Tragopan melanocephalus
Source: Animals (Basel). 2021 Mar 4;11(3):680. doi: 10.3390/ani11030680 (PMC7999559; doi:10.3390/ani11030680)
Supplement: Supplementary file 1 [file animals-11-00680-s001.zip › animals-1035086-s1.pdf]

Questionnaire for Assessment of Protected Areas Management Effectiveness in the conservation of Western Tragopan in Pakistan

Issue	Criteria	Score: Tick only one	Comments
**1. CONTEXT (where are we now?)**			
1. Legal status	The protected area is not gazetted	0	
	There is agreement that the protected area should be gazetted	1	
	The protected area is in the process of being gazetted	2	
	The protected area has been formally gazetted/covenanted	3	
2. Protected Area Regulations	There are no regulations	0	
	Regulations with major weaknesses	1	
	Regulations with some weaknesses or gaps	2	
	Regulations provide an excellent basis for management	3	
3. Protected Area boundary demarcation	The boundary of the protected area is not known	0	
	The boundary of the protected area is known by the management authority but is not known by local residents	1	
	The boundary of the protected area is known but is not demarcated	2	
	The boundary of the protected area is known and is appropriately demarcated	3	
4. Biodiversity Resource inventory	There is little or no information available on the critical habitats, species and cultural values of the protected area	0	
	Information is not sufficient to support planning and decision making	1	
	Information is sufficient for most key areas	2	
	Information is sufficient to support all areas	3	
5. Species Resource inventory	There is no information available on the target species (Western Tragopan)	0	
	Information is not sufficient to support planning and decision making on species	1	
	Information is little information available for target species	2	
	Information is sufficient information available on species	3	
**2. PLANNING (where do we want to be?)**			
1. Protected area design	Inadequacies in protected area design mean achieving the major objectives of the protected area is very difficult	0	
	Inadequacies in protected area design mean that achievement of major objectives is difficult but some mitigating actions are being taken	1	
	Protected area design is not significantly constraining achievement of objectives, but could be improved	2	
	Protected area design helps achievement of objectives; it is appropriate for species and habitat conservation	3	
2.Strategic Management plan	There is no management plan	0	
	Management plan is not being implemented	1	
	Management plans exists and is partially implemented	2	
	A management plan exists and is being implemented	3	
3.Conservation Development Framework	No Conservation Development Framework exists	0	
	Exists but few of the activities are implemented	1	
	Exists and many activities are implemented	2	
	Exists and all activities are implemented	3	
4.Species specific action plan	No Conservation action plan for the target species exists	0	
	Exists but few of the activities are implemented	1	
	Exists and many activities are implemented	2	
	Exists and all activities are implemented	3	
5. Planning outside of the protected	No Conservation action plan for the target species exists outside	0	

area for target species	PA		
	Exists but few of the activities are implemented	1	
	Exists and many activities are implemented	2	
	Exists and all activities are implemented	3	
**3. INPUTS (what do we need?)**			
1. Research & Monitoring Programme	There is no survey or research work taking place	0	
	There is a small amount of survey and research work	1	
	There is considerable survey and research work	2	
	There is a comprehensive, integrated research programme	3	
2.Staff Numbers	There are no staff	0	
	Staff numbers are inadequate	1	
	Staff numbers are below optimum	2	
	Staff numbers are adequate	3	
3. Staff training	Staff lack the skills needed for to conduct surveys of target species	0	
	Staff training and skills are low relative to the needs	1	
	Staff training and skills are adequate, but could be further improved to fully achieve the objectives	2	
	Staff training and skills are aligned with the management needs	3	
4.Current budget	There is no budget	0	
	The available budget is inadequate for basic management needs	1	
	The available budget is acceptable but could be further improved	2	
	The available budget is sufficient	3	
5.Security of budget	Wholly reliant on outside or highly variable funding	0	
	There is very little secure budget	1	
	There is a reasonably secure core budget	2	
	There is a secure budget	3	
**4. PROCESSES (how do we go about it?)**			
1. Annual plan of operation	No Annual plan of operation exists	0	
	Exists but few of the activities are implemented	1	
	Exists and many activities are implemented	2	
	Exists and all activities are implemented	3	
2. Equipment	There are little or no equipment and facilities	0	
	There are some equipment and facilities but these are inadequate	1	
	There are equipment and facilities, but still some gaps	2	
	There are adequate equipment and facilities	3	
3, Target species management	Active resource management is not being undertaken	0	
	Very few of the requirements for active management are being implemented	1	
	Many of the requirements for active management are being implemented	2	
	Requirements are being substantially or fully implemented	3	
4.HR Management	Problems with personnel management constrain the achievement of major management objectives	0	
	Problems with personnel management partially constrain the achievement of major management objectives	1	
	Personnel management is adequate to the achievement of major management objectives but could be improved	2	
	Personnel management is excellent and aids the achievement major management objectives	3	
5. Management of budget	Budget management is very poor and significantly undermines effectiveness	0	
	Budget management is poor and constrains effectiveness	1	
	Budget management is adequate but could be improved	2	
	Budget management is excellent and meets management needs	3	
6, Monitoring and evaluation	There is no monitoring and evaluation	0	
	There is some *ad hoc* monitoring and evaluation, but no overall strategy	1	
	There is an agreed and implemented monitoring and evaluation system but results do not feed back into management	2	
	A good monitoring and evaluation system exists, and is well implemented	3	
**5. OUTPUTS/OUTCOMES (what are the results / achievements?)**			
1. Ecological condition assessment	Many important biodiversity, ecological or cultural values are	0	

	being severely degraded		
	Some biodiversity, ecological or cultural values are being severely degraded	1	
	Some biodiversity, ecological and cultural values are being partially degraded but the most important values have not been significantly impacted	2	
	Biodiversity, ecological and cultural values are predominantly intact	3	
2. Species conservation status assessment	Active assessment of target species is not being undertaken	0	
	Very few of the assessment are being undertaken	1	
	Many of the assessment are being undertaken	2	
	Active assessment are being undertaken	3	
3. Species Protection systems	Available management mechanisms is not working to protect species	0	
	Available management mechanisms is poorly working to protect species	1	
	Available management mechanisms is working to protect species	2	
	Available management mechanisms is effectively working to protect species	3	
