# Supplementary material for: The Effectiveness of Protected Areas in Conserving Globally Threatened Western Tragopan Tragopan melanocephalus
Source: Animals (Basel). 2021 Mar 4;11(3):680. doi: 10.3390/ani11030680 (PMC7999559; doi:10.3390/ani11030680)
Supplement: Supplementary file 1 [file animals-11-00680-s001.zip › animals-1035086-s2.pdf]

Questionnaire for Assessment of Threats to Species and its habitat in PAs

1. Residential and commercial development within a protected area especially in species habitat
Threats from human settlements or other non-agricultural land uses with a substantial footprint

High	Medium	Low	N/A	
				1.1 Housing and settlement
				1.2 Commercial and industrial areas
				1.3 Tourism and recreation infrastructure
				1.4 Roads and paths

2. Agriculture within a protected area
Threats from farming and grazing as a result of agricultural expansion and intensification, including silviculture and aquaculture

High	Medium	Low	N/A	
				2.1 Annual and perennial non-timber crop cultivation
				2.1a Drug cultivation
				2.2 Wood and pulp plantations
				2.3 Livestock farming and grazing
				2.4 Freshwater aquaculture

3. Energy production and mining within a protected area
Threats from production of non-biological resources

High	Medium	Low	N/A	
				3.1 Oil and gas drilling
				3.2 Mining and quarrying
				3.3 Energy generation, including from hydropower dams

4. Transportation and service corridors within a protected area
Threats from long narrow transport corridors and the vehicles that use them including associated wildlife Mortality

High	Medium	Low	N/A	
				4.1 Roads and railroads (include road-killed animals)
				4.2 Utility and service lines (e.g. electricity cables, telephone lines,)
				4.3 Shipping lanes and canals
				4.4 Flight paths

5. Biological resource use and harm within a protected area
Threats from consumptive use of "wild" biological resources including both deliberate and unintentional harvesting effects; also persecution or control of specific species (note this includes hunting and killing of animals)

High	Medium	Low	N/A	
				5.1 Hunting, killing and collecting terrestrial animals (including killing of animals as a result of human/wildlife conflict)
				5.2 Gathering terrestrial plants or plant products (non-timber)
				5.3 Logging and wood harvesting
				5.4 Fishing, killing and harvesting aquatic resources

6. Human intrusions and disturbance within a protected area
Threats from human activities that alter, destroy or disturb habitats and species associated with nonconsumptive uses of biological resources

High	Medium	Low	N/A	
				6.1 Recreational activities and tourism
				6.2 War, civil unrest and military exercises
				6.3 Research, education and other work-related activities in protected areas
				6.4 Activities of protected area managers (e.g. construction or vehicle use, artificial watering points and dams)
				6.5 Deliberate vandalism, destructive activities or threats to protected area staff and visitors

7. Natural system modifications
Threats from other actions that convert or degrade habitat or change the way the ecosystem functions

High	Medium	Low	N/A	
				7.1 Fire and fire suppression (including arson)
				7.2 Dams, hydrological modification and water management/use
				7.3a Increased habitat fragmentation within protected area
				7.3b Isolation from other natural habitat (e.g. deforestation, dams without effective aquatic wildlife passages)
				7.3c Other ‘edge effects’ on park values
				7.3d Loss of keystone species (e.g. top predators, pollinators etc)

8. Invasive and other problematic species and genes
Threats from terrestrial and aquatic non-native and native plants, animals, pathogens/microbes or genetic materials that have or are predicted to have harmful effects on biodiversity following introduction, spread and/or increase

High	Medium	Low	N/A	
				8.1 Invasive non-native/alien plants (weeds)
				8.1a Invasive non-native/alien animals
				8.1b Pathogens (non-native or native but creating new/increased problems)
				8.2 Introduced genetic material (e.g. genetically modified organisms)

9. Pollution entering or generated within protected area
Threats from introduction of exotic and/or excess materials or energy from point and non-point sources

High	Medium	Low	N/A	
				9.1 Household sewage and urban waste water
				9.1a Sewage and waste water from protected area facilities (e.g. toilets, hotels etc)
				9.2 Industrial, mining and military effluents and discharges (e.g. poor water quality discharge from dams, e.g. unnatural temperatures, de-oxygenated, other pollution)
				9.3 Agricultural and forestry effluents (e.g. excess fertilizers or pesticides)
				9.4 Garbage and solid waste

				9.5 Air-borne pollutants
				9.6 Excess energy (e.g. heat pollution, lights etc)

10. Geological events

Geological events may be part of natural disturbance regimes in many ecosystems. But they can be a threat if a species or habitat is damaged and has lost its resilience and is vulnerable to disturbance. Management capacity to respond to some of these changes may be limited.

High	Medium	Low	N/A	
				10.1 Volcanoes
				10.2 Earthquakes/Tsunamis
				10.3 Avalanches/ Landslides
				10.4 Erosion and siltation/ deposition (e.g. shoreline or riverbed changes)

11. Climate change and severe weather

Threats from long-term climatic changes which may be linked to global warming and other severe climatic/ weather events outside of the natural range of variation

High	Medium	Low	N/A	
				11.1 Habitat shifting and alteration
				11.2 Droughts
				11.3 Temperature extremes
				11.4 Storms and flooding

12. Specific cultural and social threats

High	Medium	Low	N/A	
				12.1 Loss of cultural links, traditional knowledge and/or management practices
				12.2 Natural deterioration of important cultural site values etc
				12.3 Destruction of cultural heritage buildings, gardens, sites
